# YPR2 is a regulator of light modulated carbon and secondary metabolism in *Trichoderma reesei*

**DOI:** 10.1186/s12864-019-5574-8

**Published:** 2019-03-13

**Authors:** Eva Hitzenhammer, Christoph Büschl, Michael Sulyok, Rainer Schuhmacher, Bernhard Kluger, Elisabeth Wischnitzki, Monika Schmoll

**Affiliations:** 1 AIT - Austrian Institute of Technology GmbH, Center for Health and Bioresources, Konrad-Lorenz-Strasse 24, 3430 Tulln, Austria; 2 Department of Agrobiotechnology (IFA-Tulln), Center for Analytical Chemistry, University of Natural Resources and Life Sciences Vienna, (BOKU), Konrad-Lorenz-Straße 20, 3430 Tulln, Austria

**Keywords:** *Trichoderma reesei*, *Hypocrea jecorina*, Secondary metabolism, Alamethicin, Carbon metabolism, Light response

## Abstract

**Background:**

Filamentous fungi have evolved to succeed in nature by efficient growth and degradation of substrates, but also due to the production of secondary metabolites including mycotoxins. For *Trichoderma reesei*, as a biotechnological workhorse for homologous and heterologous protein production, secondary metabolite secretion is of particular importance for industrial application. Recent studies revealed an interconnected regulation of enzyme gene expression and carbon metabolism with secondary metabolism.

**Results:**

Here, we investigated gene regulation by YPR2, one out of two transcription factors located within the SOR cluster of *T. reesei*, which is involved in biosynthesis of sorbicillinoids. Transcriptome analysis showed that YPR2 exerts its major function in constant darkness upon growth on cellulose. Targets (direct and indirect) of YPR2 overlap with induction specific genes as well as with targets of the carbon catabolite repressor CRE1 and a considerable proportion is regulated by photoreceptors as well. Functional category analysis revealed both effects on carbon metabolism and secondary metabolism. Further, we found indications for an involvement of YPR2 in regulation of siderophores. In agreement with transcriptome data, mass spectrometric analyses revealed a broad alteration in metabolite patterns in ∆*ypr2*. Additionally, YPR2 positively influenced alamethicin levels along with transcript levels of the alamethicin synthase *tex1* and is essential for production of orsellinic acid in darkness.

**Conclusions:**

YPR2 is an important regulator balancing secondary metabolism with carbon metabolism in darkness and depending on the carbon source. The function of YPR2 reaches beyond the SOR cluster in which *ypr2* is located and happens downstream of carbon catabolite repression mediated by CRE1.

**Electronic supplementary material:**

The online version of this article (10.1186/s12864-019-5574-8) contains supplementary material, which is available to authorized users.

## Background

In their natural environment, fungi can only succeed by efficient distribution of resources between the tasks of substrate degradation and growth, reproduction and fending off competitors, often by chemical warfare [1, 2]. Accordingly, they balance primary and secondary metabolism depending on the situation in their natural habitat. Regulation of secondary metabolism occurs in response to diverse environmental signals including nutrient abundance and quality as well as light [3]. Recent studies indicate that fungi control the switch between primary and secondary metabolism [4] and that enzyme production, carbon catabolite repression and secondary metabolite production are interlinked processes [4, 5]. Moreover, there are indications that this switching does also involve an adjustment to light and darkness as well as nutritional conditions [5, 6].

**Fig. 1:**
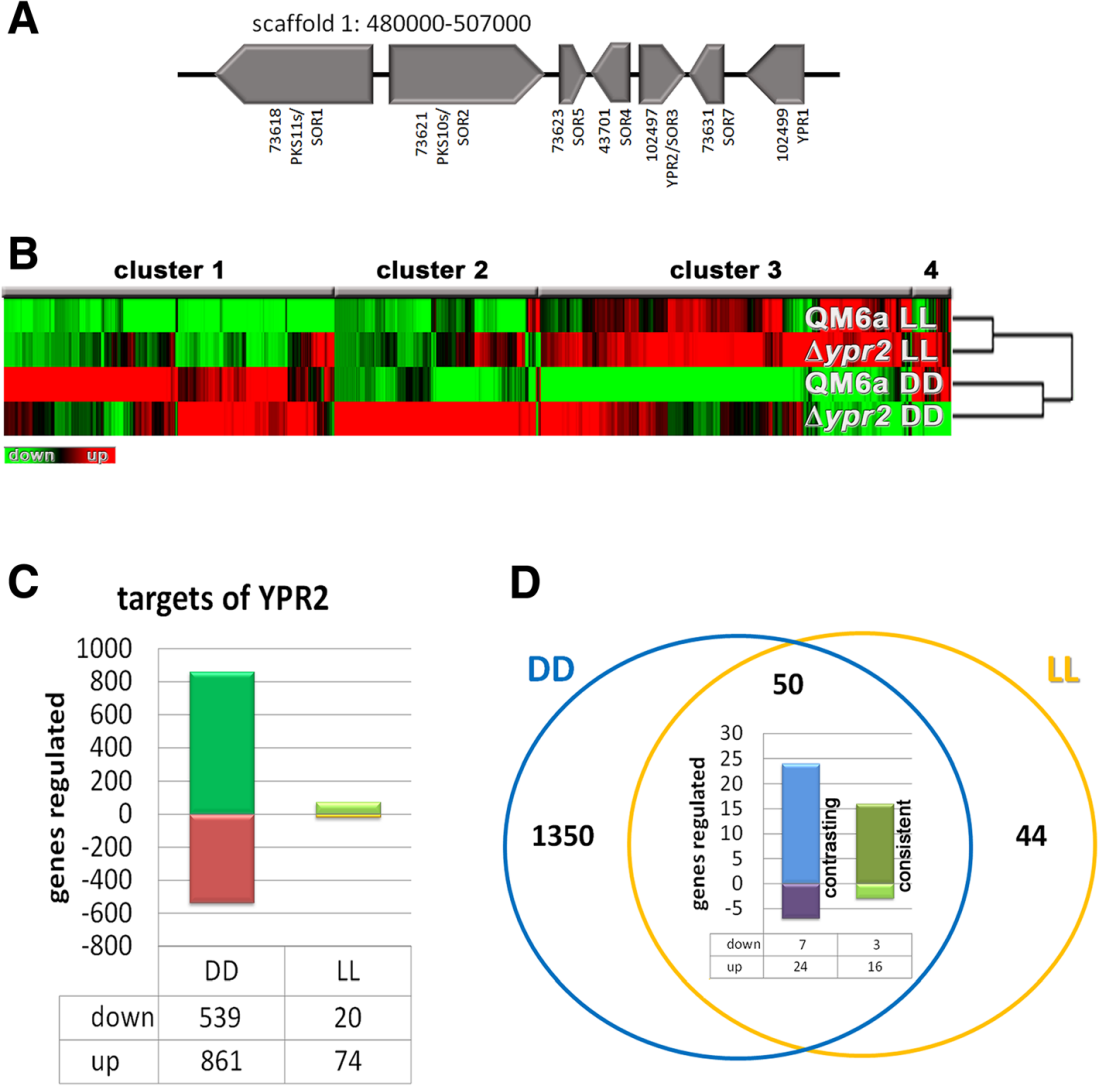
Gene regulation by YPR2 in *T. reesei*. **a** Schematic representation of the SOR cluster. Genomic locations are taken from the JGI Trichoderma reesei database v2.0 (https://genome.jgi.doe.gov/Trire2/Trire2.home.html). **b** Hierarchical clustering of gene regulation patterns in ∆*ypr2* compared to wildtype in constant light (LL) and constant darkness (DD) upon growth on cellulose. **c** Numbers of genes regulated in ∆*ypr2* in constant light or constant darkness on cellulose (≥2fold, *p*-value threshold 0.01). **d** Genes directly or indirectly regulated by YPR2 in constant light overlapping with gene regulation by YPR2 in constant darkness. The diagram shows the proportion of consistent regulation (upregulation in ∆*ypr2* in light and darkness, downregulation in light and darkness) or contrasting regulation (upregulation in light and downregulation in darkness (“up”) or downregulation in light and upregulation in darkness (“down”))

*Trichoderma reesei* is a filamentous ascomycete which is frequently applied in industry for homologous and heterologous protein production, most importantly cellulases [7]. Therefore, this fungus has also become a model for enzymology and regulation of plant cell wall degradation [8, 9]. Degradation of compounds constituting plant biomass, especially cellulose, is regulated by several transcription factors [10] and in response to the nutrient source available [11]. The most important inducing carbon sources are cellulose and lactose, while glucose represses cellulase gene expression by CRE1 mediated carbon catabolite repression [12]. Comparison of gene regulation on inducing versus repressing carbon sources yielded a gene set specific for inducing conditions in *T. reesei* [13]. Besides nutrient components, in recent years also light emerged as a crucial environmental cue influencing modulation of cellulase gene expression and enzyme production in general in *T. reesei* [14, 15]. Light considerably impacts the physiology of fungi [16, 17] and also of *T. reesei* in several ways. Conidiation is enhanced in light in *T. reesei* and for sexual development light-dark cycles are the preferred condition [18]. Moreover, growth on plates and in liquid culture is altered in light compared to darkness [15, 19, 20]. The photoreceptors BLR1, BLR2 and ENV1 play important roles in cellulase regulation [18, 19]. Genome wide analysis in *T. reesei* and *N. crassa* showed that regulation of CAZyme (carbohydrate active enzymes) gene expression by photoreceptors is a conserved function [21, 22]. Based on the findings of the relevance of light, also transcriptome analysis of the function of CRE1 upon growth on cellulose was performed under controlled light conditions. The respective analysis showed that CRE1 regulates the SOR cluster (Fig. 1a) positively in darkness and negatively in light [5]. Besides the biosynthetic genes, the SOR cluster also comprises the transcription factor YPR2. The sorbicillin derivatives dihydrotrichotetronin and trichodimerol are the major components biosynthesized with involvement of the SOR cluster [5].

The group of sorbicillinoids contains various yellow pigments and different compounds are produced by filamentous fungi such as *Trichoderma*, *Aspergillus*, *Verticillium* and *Penicillium* [23–26]. They show pharmaceutically valuable activities as they have been found to act as antivirals, anti-inflammatories and antimicrobials with potential applications for treatment of HIV and even cancer (for an overview see [26] and references therein). Interestingly, the cluster responsible for production of sorbicillin derivatives, as identified in *Penicillium chrysogenum* [26] is conserved in *T. reesei*, but not in other closely related fungi [5, 24]. The hypothesis that *Trichoderma* spp. may have acquired gene clusters due to their evolutionary origin as mycoparasites [27, 28] suggests that horizontal gene transfer (HGT) is likely responsible for this unequal distribution within Sordariomycetes.

In *T. reesei* the production of metabolites is particularly critical due to its status as a GRAS organism [29] and its application as producer of homologous and heterologous proteins [30]. Consequently, the presence of potentially harmful metabolites increases costs of strain development and downstream processing in industrial fermentations. Our recent research revealed that besides paracelsins, *T. reesei* also produces the potentially pharmaceutically relevant sorbicillin derivatives dihydrotrichotetronine and trichodimerol upon growth on cellulose. This production is strongly modulated by light [5]. Within the SOR cluster, the two polyketide synthases SOR1 and SOR2 are required for the biosynthesis of these compounds, with a closely located monooxygenase and a transporter being involved in regulation of sorbcillin levels as well [5]. The transcription factor YPR1 is required for production of the yellow pigments, which were reported as sorbicillins [24], formed by *T. reesei* as well as most genes of the corresponding cluster upon growth on glucose. In contrast, YPR2 negatively influences the genes of the cluster including *ypr1* on glucose [24]. However, upon growth on cellulose, YPR2 has a positive effect on transcript levels of all cluster genes [5]. Consequently, YPR1 and YPR2 likely have carbon source dependent functions, balancing carbon and secondary metabolism. *ypr2* is down-regulated in response to light on cellulose and subject to regulation by photoreceptors [22, 31]. Additionally, *ypr2* is negatively regulated by CRE1 in light, but positively in darkness [5] and shows enhance transcript levels under conditions favouring sexual development compared to growth on cellulose [32]. Interestingly, the three biosynthetic genes within the SOR cluster show a light dependent positive feedback loop which may involve intracellular sensing of biosynthetic building blocks of sorbicillins [5]. Since this study indicated an influence not only on production of trichodimerol and dihydrotrichotetronine, but also on other secondary metabolites, we were interested how broad the regulatory effect of YPR2 is.

In this study we investigated the transcriptome as altered by YPR2 compared to wildtype upon growth on cellulose in light and darkness. We found that YPR2 exerts its function predominantly in darkness and targets both carbon and secondary metabolism. Additionally genes directly or indirectly regulated by YPR2 overlap in part with those regulated by the carbon catabolite repressor CRE1. Under the tested in vitro conditions, YPR2 influences production of secondary metabolites broadly, positively regulates alamethicin levels and is essential for production of orsellinic acid.

## Results and discussion

### YPR2 has its major function in darkness

In order to evaluate the genome wide regulatory function of YPR2 we cultivated ∆*ypr2* and wildtype on minimal medium with cellulose as carbon source under controlled light conditions for comparative transcriptome analysis. Transcript levels in the mutant strain were compared to wildtype for light and darkness separately in order to assess distinct regulation patterns by YPR2 under both conditions (>2fold regulation, *p*-value threshold 0.01). We found that the main regulatory function of YPR2 happens in darkness (Fig. 1b, c). This finding is in accordance with earlier results on global regulation of secreted metabolites by high performance thin layer chromatography (HPTLC), showing a clear alteration in ∆*ypr2* in darkness [5].

In darkness, we found 539 genes to be downregulated and 861 genes to be up-regulated in ∆*ypr2* upon growth on cellulose, while in light only 20 genes were downregulated and 74 genes upregulated (Fig. 1c, Additional file 1). Fifty genes were regulated by YPR2 in light and darkness (Fig. 1d).

Previously, we evaluated which gene set would be regulated under conditions causing cellulase induction (growth on cellulose, lactose and sophorose) compared to conditions repressing cellulase expression (glucose, glycerol), which revealed 1324 genes, we called “induction specific” [13]. We checked for a possible overlap of this gene set with that influenced by YPR2. In darkness 141 of the genes regulated by YPR2 were previously found to show induction specific regulation [13]. Although the photoreceptor proteins BLR1, BLR2 and ENV1 exert their main function in light, they influence gene regulation in darkness as well [18, 22, 33]. Interestingly, 977 genes (70%) targeted by YPR2 in darkness are subject to regulation by one or more photoreceptors [22] (Additional file 1) indicating that many of the genes influenced by YPR2 are relevant for light response as well. Transcript patterns of SOR cluster genes in this transcriptome dataset on YPR2 are in accordance with detailed RT-qPCR data shown previously [5], hence validating the presented results. Additionally, deletion of *ypr2* causes decreased transcript levels of *ypr1* (TR_104299), a strong regulator of the SOR cluster [24]. We then tested for non-random distribution of genes regulated by YPR2 in light and darkness and considered three or more neighbouring, coregulated genes as a cluster. Thereby, we detected 40 clusters upregulated in ∆*ypr2* in darkness and 30 clusters downregulated. In many cases these clusters included CAZyme encoding genes and secondary metabolism associated genes (Additional file 1). Only one such cluster was found in light.

### YPR2 impacts regulation of carbon and secondary metabolism

**Fig. 2:**
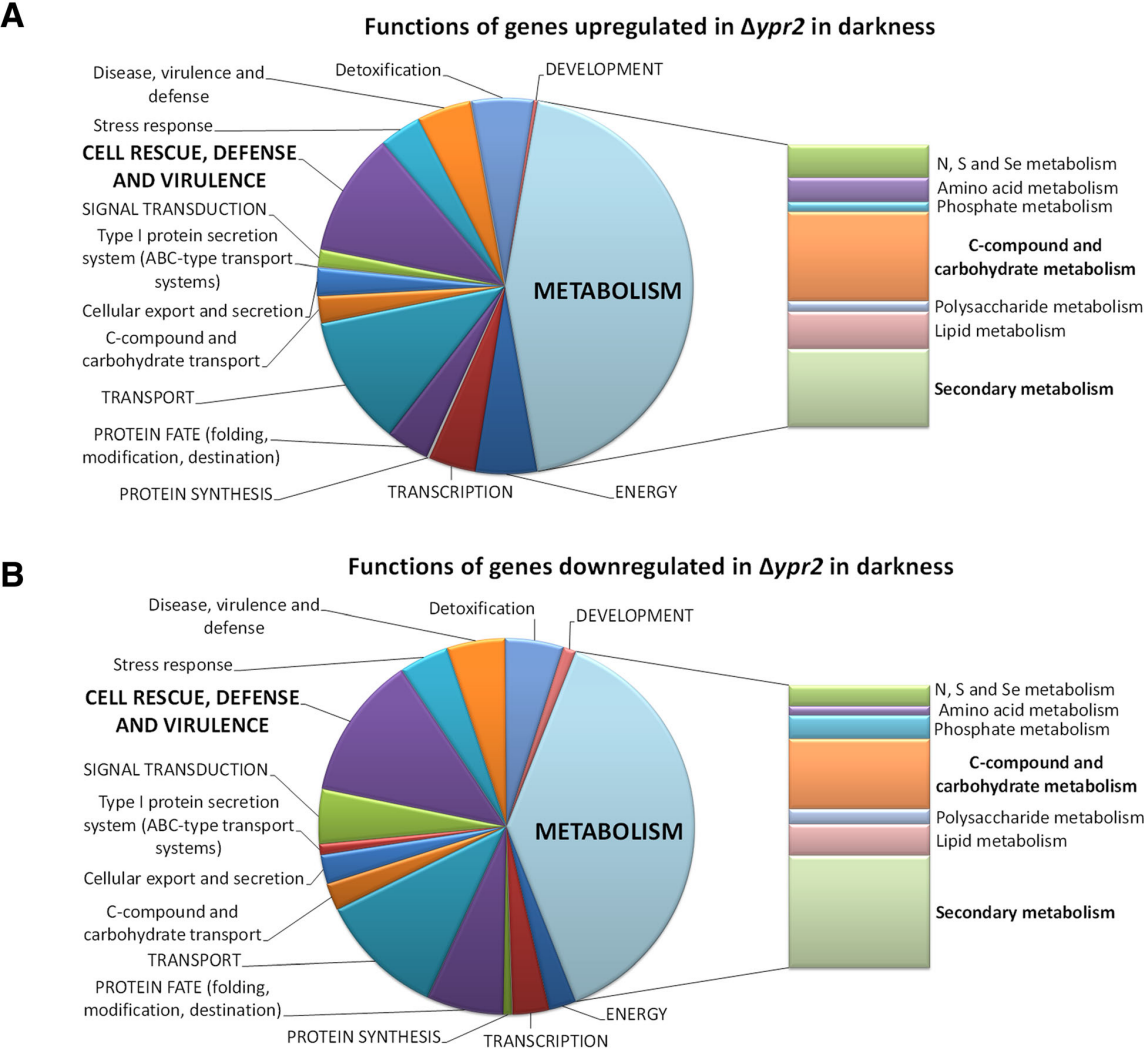
Schematic representation of functional category analysis. **a** Funcat analysis of genes up- regulated in ∆*ypr2* in darkness. **b** Funcat analysis of genes downregulated in ∆*ypr2* in darkness. For funcat overview in light see Additional file 3: Figure S1

Functional category analysis was performed to evaluate statistically significant enrichment (*p*-value < 0.05) of gene functions in the respective groups (Fig. 2a and b, Additional file 2 and Additional file 3: Figure S1). Interestingly, although numerous genes associated with metabolic functions were downregulated in darkness in ∆*ypr2*, significant enrichment was only observed for genes involved in secondary metabolism (*p*-value 5.87E-09). Specifically, enrichment occurred with metabolism of polyketides and non ribosomal peptide synthesis. Additionally, functions in siderophore-iron transport along with other transport function and correspondingly, homeostasis of metal ions as well as serine/threonine protein kinase functions were enriched.

Genes up-regulated in darkness in ∆*ypr2* showed significant enrichment in metabolic functions (*p*-value 1.29E-05), particularly in amino acid metabolism as well as regulation of nitrogen, sulphur and selenium metabolism. Moreover, genes involved in C-2, C-4 and organic acid metabolism were enriched as well as those functioning in aliphatic hydrocarbon catabolism. Enrichment of the upregulated gene set in secondary metabolism, particularly metabolism of polyketides, alkaloides and secondary products derived from L-tryptophan, L-phenylalanine and L-tyrosine indicates that lack of YPR2 in the genome causes a shift in secondary metabolite production in darkness that may involve amino acid derived compounds. Moreover, this analysis reflects a broad impact of YPR2 on carbon and secondary metabolism (Fig. 2a and b).

Enrichment of glycolysis and gluconeogenesis related genes among those upregulated in darkness indicates increased investment of resources upon lack of YPR2, which might be fueled by enrichment of genes with functions in C-compound and carbohydrate transport. Interestingly, also genes involved in stress response show significant enrichment in upregulated genes in darkness, including catalase functions and particularly strong enrichment in detoxification functions.

The numbers of genes regulated by YPR2 in light are considerable smaller than in darkness. Among genes down regulated in light in ∆*ypr2,* genes involved in secondary metabolism are enriched as well along with different functions in transport. Upregulated genes in light are enriched in C-compound and carbohydrate metabolism, polysaccharide metabolism as well as transport facilities. Again, as seen in darkness, the enrichment in functions in secondary metabolism in up- and down regulated genes also in light indicates that the functional shift as observed in darkness, occurs.

### Genes regulated by YPR2 in darkness

A total of 61 CAZyme encoding genes are upregulated in ∆*ypr2*, including 15 carbohydrate esterase genes, 38 glycoside hydrolase genes of diverse families and six glycosyl transferase genes (Additional file 1). Among these genes are four chitinases including *ech42* and *chit36*, which are involved in mycoparasitism, extracellular chitin degradation and recycling of cell wall components upon autolysis and starvation [34–36]. Moreover, the alpha-galactosidase genes *agl1* and *agl2* as well as *lxr1* encoding a mannitol dehydrogenase [37] are upregulated in ∆*ypr2* in darkness. The heterotrimeric G-protein pathway has been shown to function in sexual development [38], regulation of cellulase gene expression [14] and glucose sensing [13] in *T. reesei* and diverse functions in other fungi [39]. Of the 57 G-protein coupled receptors of *T. reesei* [9], 11 are up-regulated in ∆*ypr2* including the pheromone receptor gene *hpr1* and the peptide pheromone transporter gene *ste6p*. Additionally the meiosis related genes *ecm4*, *pdc1*, *gtt1* and *msc1* were up-regulated. However, no alterations in sexual development were observed for ∆*ypr2* (E. Stappler, unpublished).

Concerning secondary metabolism, we found the regulator *vel1*, which is involved in chemical communication upon sexual development [40] as well as in cellulase regulation [41] to be up-regulated in ∆*ypr2* along with 11 genes encoding cytochrome P450 proteins, the NRPS gene *tex19*, the PKS/NRPS hybrid gene *tex11* and the PKS gene *pks9g*, for which no functional characterization is available.

The strikingly high number of 59 transcription factor genes positively influenced by YPR2 suggests a flat hierarchical regulatory network triggered by YPR2. Unfortunately, none of these transcription factor genes has been studied in detail so far.

Of the seven catalase genes detected in *T. reesei* [9], 4 are upregulated in darkness in ∆*ypr2* up to more than 20 fold indicating a strong antioxidant response balanced by YPR2.

Among the genes downregulated in ∆*ypr2* we found 30 CAZyme encoding genes, including numerous carbohydrate esterase genes, glycoside hydrolases and glycosyl transferases (Additional file 1). However, as with up-regulated genes, the classical genes required for plant cell wall degradation, particularly cellulases and hemicellulases are not the targets of YPR2 and neither are the known cellulase transcription factors. Only *vib1*, which was recently shown to be involved in cellulase regulation in *T. reesei* [42] and *N. crassa* [43] is a target of YPR2 with transcript levels decreased by roughly 60% in darkness (Additional file 1).

The downregulated gene set associated with secondary metabolism (14 genes) includes 5 genes encoding cytochrome P450 proteins, the putative alamethicin synthase *tex1* and several more pks and terpenoid synthase genes.

Down-regulation of 9 G-protein coupled receptors, while also several GPCRs are up-regulated in the absence of YPR2, indicates a shift in priorities of signal perception triggered by YPR2.

Interestingly, the hydrophobin genes *hfb1*, *hfb2*, *hfb3* and *hfb5* as well as *epl1*/*sm1* were downregulated in ∆*ypr2*. Known functions of hydrophobins include many morphogenetic events like sexual (fruiting body formation) and asexual development (sporulation) as well as infection structure formation [44]. An antioxidant activity of *T. reesei* hydrophobins was suggested by a recent study [45]. The ceratoplatanin elicitor Sm1 is important for plant root interaction and elicitation of disease resistance by *Trichoderma* spp. [46, 47], while its *Sclerotinia sclerotiorum* homologue is relevant for pathogenicity [48]. These biological roles may be connected to genes regulated by YPR2 targeting sexual development, signaling and secondary metabolism due to an effect on chemical communication and interaction with fungi and plants in the environment.

Not only fold-regulation, but also absolute transcript levels are relevant as they reflect an investment of considerable resources for expression of a given gene. Therefore we checked for striking alterations among the 100 genes with highest detected transcript levels in ∆*ypr2* compared to wildtype. The GMC oxidoreductase gene encoding *aox1* was among the 10 genes with the strongest signal in the mutant in contrast to wildtype, with 25fold upregulation in ∆*ypr2.* Interestingly, *aox1* is also strongly upregulated in ∆*cre1* in darkness [5]. Additionally, a gene encoding an extracellular membrane protein (TR_123475) and a gene encoding a small cystein rich protein (TR_105533), both with potential effector function as well as a transporter with putative putative tetracyclin resistance function (TR_44956) and a gene of unknown function (TR_44967) show high transcript abundance in ∆*ypr2*, but not wildtype.

### Genes regulated by YPR2 in light

Compared to the effect of YPR2 in darkness, only few genes are directly or indirectly regulated by YPR2 in light (Fig. 1c). Interestingly, in contrast to darkness, upregulation was detected for several genes encoding plant cell wall degrading enzymes. However, transcript levels of these genes in QM6a is at very low levels and even hardly detectable in some cases in light on cellulose and the increase (albeit considerable in fold values) in ∆*ypr2* does by far not reach darkness levels of these transcripts. Essentially the same applies also to the putative lactose permease TR_3405 [49], which is upregulated in ∆*ypr2* in light, but expressed at considerably higher levels in darkness.

TR_121251 encoding a putative effector protein [9] is upregulated in light in ∆*ypr2.* The encoded protein is related to the Mad1 adhesin of *Metarrhizium anisopliae* [50], which is relevant for adhesion and germination.

### Consistent and contrasting regulation by YPR2 in light and darkness

Of the genes consistently upregulated in light and darkness in ∆*ypr2* (Fig. 1d), TR_74282 encoding a QID74 homologue is particularly interesting. While about 3.7 fold upregulated in light, it is more than 28fold upregulated in darkness, thereby being the most highly expressed gene in ∆*ypr2* in darkness*.* In *T. harzianum* the cell wall protein QID74 is strongly expressed during starvation and was shown to be relevant cell wall protection and adherence to hydrophobic surfaces. Heterologous expression in yeast further suggested a function in mating and sporulation [51]. Additionally, QID74 was shown to impact plant root architecture upon association with *T. harzianum* [52]. Together with the regulation of hydrophobin genes, GPCRs and secondary metabolism by YPR2 a function in regulation of pathways important for association with plants in nature would be conceivable.

Analyzing the genes misregulated in ∆*ypr2* (including direct and indirect targets) in light and darkness we noted that in many cases the effect of YPR2 in light was the opposite of that in darkness (Fig. 1d). Therefore we wanted to check for a functional relevance of such a light dependent effect of YPR2. Besides TR_43701 encoding SOR4, the multidrug transporter of the SOR cluster [5], several other as yet uncharacterized genes showed contrasting regulation in light and darkness by YPR2.

Intriguingly, we found also a coregulated siderophore cluster located on chromosome 5 ([53]; genes 1083–1088)/scaffold 46 (26764–44,919) [8], which is conserved in *Aspergillus fumigatus*. It comprises the genes encoding homologues of the NRPS SidD (TR_71005), the transacylase SidF (TR_82628), the siderophore biosynthesis lipase/esterase SidJ involved in siderophore hydrolysis (TR_112590), the ABC multidrugtransporter SitT (TR_71010), the hydroxyornithine transacylase SidF (TR_82628), the enoyl-CoA hydratase/isomerase family protein sidH (TR_6085) and the siderophore iron transporter MirB (TR_71008). Fusarinin that is expected to be produced by the proteins encoded in this cluster [54] was found previously to be produced in *T. reesei* QM6a [55].

This cluster is in the wildtype differentially regulated in light and darkness. It is consistently downregulated in ∆*ypr2* in darkness and up-regulated in ∆*ypr2* in light suggesting light specific regulation of siderophore production by YPR2. The high affinity iron uptake system employing siderophores is particularly important under iron limited conditions [56]. Therefore we checked if deletion of YPR2 might cause a general misbalance in iron sensing and uptake systems at the transcriptional level.

Reductive iron assimilation (RIA) represents another high affinity iron uptake system [56] and is represented in *T. reesei* by two Fet3-homologues, the multicopperoxidases TR_102820 (FET3a) and TR_5119 (FET3b), and two Ftr1 homologues, the high affinity iron permeases TR_54962 (FTR1a) and TR_80639 (FTR1b). FET3a and FTR1a (scaffold 1: 1684330–1,690,370) as well as FET3b and FTR1b (scaffold 1:561024–565,836) are located next to each other and appear to share a bidirectional promotor. *fet3a* and *ftr1a* are coregulated and show increased transcript levels in light, but no regulation by YPR2. *fet3b* and *ftr1b* are downregulated in light, and *ftr1b* shows a similar regulation as the siderophore cluster being downregulated in ∆*ypr2* in darkness and upregulated in ∆*ypr2* in light. Consequently, YPR2 impacts regulation of one of two high affinity iron permeases, although we cannot exclude that the altered transcript levels of *ftr1b* are due to indirect regulation and caused by altered siderophore availability.

TR_4231 encoding a homologue of the *Aspergillus fumigatus* siderophore biosynthesis repressor SreA [54] is upregulated in darkness in ∆*ypr2.* The homologue of the negative regulator of SreA, HapX (TR_77191), which is negatively influenced by increasing iron levels, is not a target of YPR2.

Despite the striking regulation patterns in our data, regulation of the iron uptake systems could also be due to different growth rates between wildtype and mutant strain and hence altered iron consumption/availability. In darkness, biomass formation of ∆*ypr2* is indeed decreased compared to wildtype (to 16.4% ± 1.9%). However, in light biomass formation of wildtype and ∆*ypr2* are not significantly different, but the cluster still becomes upregulated, indicating that regulation by YPR2 and not merely altered biomass formation is the reason for the difference. Upregulation of *sreA* in ∆*ypr2* in darkness would be in accordance with a reaction to higher iron availability because of lower biomass formation. Nevertheless, regulation of the FET3 and FTR1 homologues as well as of the HapX homologue is not consistent with a hypothesis of regulation of the siderophore cluster solely due to altered iron availability and biomass formation.

A decrease in oxidative stress resistance of siderophore mutants is attributed to an iron limitation, which would be required for several oxidative stress detoxifying enzymes like catalases. Upon deletion of *ypr2*, 4 catalases are upregulated in darkness which would not contradict this hypothesis, although it remains to be confirmed whether the requirement of iron impacts catalase regulation at the transcriptional level or merely at the activity level.

### Regulatory overlap with CRE1 targets

The carbon catabolite repressor CRE1 was shown to regulate *ypr2* along with the SOR cluster negatively in light and positively in darkness [5]. Consequently we were interested in investigating if CRE1 and YPR2 share regulatory targets, which would then be subject to a double lock mechanism.

**Fig. 3:**
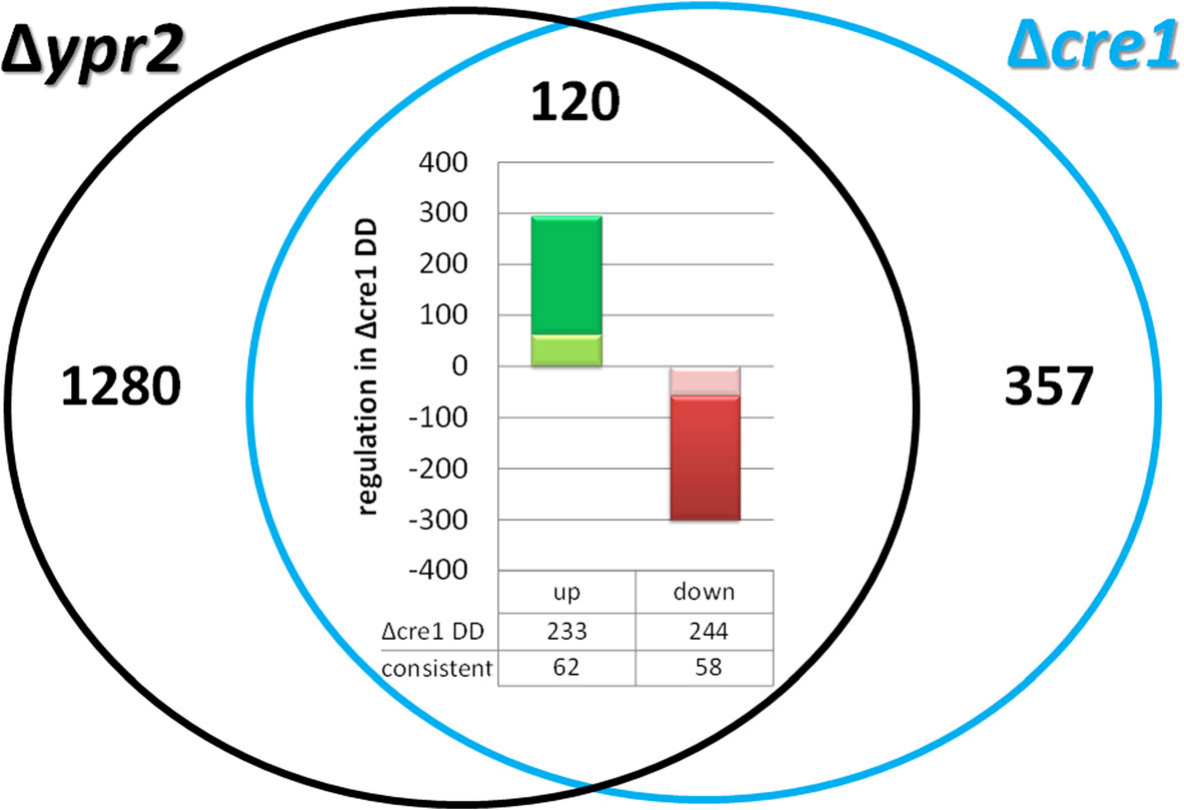
Comparison of gene regulation by YPR2 in darkness with targets (direct or indirect) of CRE1. Amount of genes regulated in ∆*ypr2* in constant darkness compared to wildtype versus those regulated in ∆*cre1* in darkness. In ∆*cre1* 233 genes are upregulated in constant darkness and 244 genes are downregulated in constant darkness [5]. Of the 447 genes regulated by CRE1 in darkness, 62 are consistently upregulated in both mutant strains (light green area) and 58 are consistently downregulated in both mutants. In total, of the 447 genes regulated by in ∆*cre1* in darkness, 120 are consistently regulated in ∆*ypr2* suggesting a double lock mechanism for these genes

Interestingly, among the 1402 genes regulated by YPR2 in darkness, we found 262 gene regulated by CRE1 either in light or darkness (Fig. 3; Additional file 1). In many cases, we observed contrasting regulation by YPR2 and CRE1 (upregulation by YPR2 and downregulation by CRE1 or vice versa). Consistent regulation by YPR2 and CRE1 was detected for 120 genes, with 58 genes positively regulated by CRE1 and YPR2 and 62 genes consistently negatively regulated by both (double lock mechanism). The gene set of up-regulated genes in both mutant strains compared to the wildtype strain comprises several genes involved in carbon and secondary metabolism and showed enrichment in functions in amino acid metabolism (*p*-value 8.58e-04) and glycolysis and gluconeogenesis (*p*-value 3.61e-03).

The consistently upregulated genes include the two transcription factors TR_72611 and TR_102920. TR_72611 is related to *Fusarium solani* CTF1B, the cutinase transcription factor 1beta, which activates cutinase genes [57]. The consistently downregulated genes include the transcription factors PRO1 (TR_76590) and TR_121682. PRO1 acts as a master regulator of signaling genes involved in development and also targets the cell wall integrity MAPkinase pathway [58], which was reported to regulate cellulase gene expression in *T. reesei* [59].

Hence the overlap of YPR2 targets with those of CRE1 in metabolic functions suggests that these transcription factors act in part in the same cascade. CRE1 regulates transcript levels of *ypr2* [5], but YPR2 does not influence *cre1* levels. Together with the differential regulation of the SOR cluster genes by YPR2 on glucose and cellulose [5, 24], we conclude that YPR2 acts downstream of carbon catabolite repression.

### YPR2 impacts biosynthesis of alamethicin and orsellinic acid

**Fig. 4:**
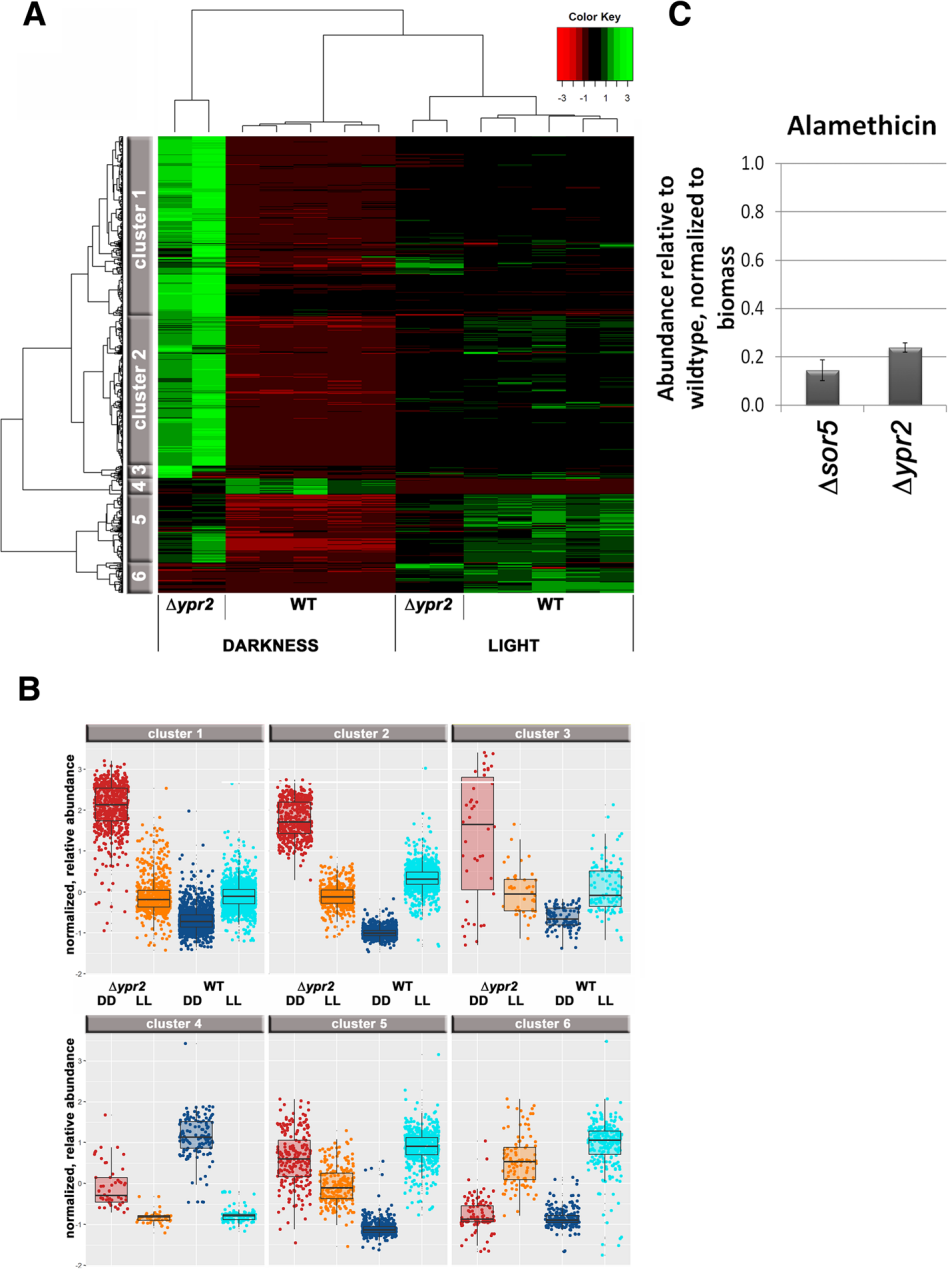
Secondary metabolite production in ∆*ypr2* upon growth on cellulose. **a** Results from mass spectrometric analysis revealed 6 clusters of regulation patterns. **b** Box plots show levels within the clusters as normalized to biomass formation. Mostly, biosynthesis level even decrease below wildtype in the dark. For smaller sets (cluster 5) elevated levels were observed in the mutant compared to wildtype. **c** Abundance of Alamethicine in samples lacking *sor5* (TR_73623) and *ypr2* (TR_102497) upon growth on minimal media with cellulose as carbon source, relative to QM6a and normalized to the biomass produced under these conditions. Errorbars indicate standard deviations of at least two biological replicates

Previous data indicated that the regulatory function of YPR2 is not limited to the SOR cluster, as besides trichodimerol and dihydrotrichotetronine, also paracelsin B levels decreased in a *ypr2* mutant strain [5]. Therefore we performed mass spectrometry analysis on cultures grown under the same conditions as for transcriptome analysis (Additional file 4). We found 6 clusters of secondary metabolite profiles obtained for the culture supernatants, which show the light-dependent involvement of YPR2 in the underlying metabolic processes (Fig. 4a). In agreement with transcriptome data, the major differences between wildtype and ∆*ypr2* can be seen upon cultivation in darkness (Fig. 4b).

Our transcriptome data clearly confirmed regulation of the SOR cluster genes by YPR2 (Additional file 1) as shown previously [5]. Surprisingly, the predicted paracelsin synthase, the NRPS TR_123786 [60] is not regulated by YPR2 and although paracelsin B levels are strongly decreased in light in ∆*ypr2* [5], transcript abundance of TR_123786 increases in light in both the wildtype and in ∆*ypr2*. As coregulation of genes indicates a regulatory relationship, we checked for coregulated genes with *ypr2* under conditions known to be relevant for secondary metabolism (different carbon sources, light/photoreceptors). We chose regulation on cellulose, glucose, lactose, glycerol and sophorose in light and darkness (dataset from [13]) as well as in photoreceptor mutants in light and darkness (dataset from [22]). Comparison showed one consistently coregulated NRPS gene, TR_60751, which is however related to a ferrichrome synthase and supports the relevance of YPR2 for siderophore regulation rather than a function in paracelsin production. We conclude that the regulatory effect of YPR2 on paracelsin levels is indirect and does not occur on the transcriptional level.

Our findings on regulation rather indicate that higher order regulation mechanisms should be considered. One such mechanism would be regulation by upstream open reading frames (uORFs), which could interfere with translation of the downstream target ORF [61]. Several short exons at the start of the predicted ORF of TR_123786 encoding a predicted paracelsin synthase could indeed represent such uORFs. Since no characterized homologues of TR_123786 are available from other fungi, clarification of the regulation mechanism of paracelsin biosynthesis warrants further detailed investigations.

A targeted screening by a mass spectrometry approach using a standardized method and internal standard compounds revealed the regulation of alamethicin biosynthesis by YPR2 in darkness on cellulose (Fig. 4c). Alamethicin was previously reported to be produced by *Trichoderma* spp. [62], albeit only by those species of the brevicompactum clade [63]. Alamethicin is reported to permeabilize Arabidopsis seedlings, which can be counteracted by prior treatment with cellulase [64]. These findings on a relevance of alamethicin in plant interaction are in agreement with both a carbon source depending function of YPR2: the function of YPR2 on glucose [24] is different to that on cellulose [5]. As cellulase regulation also happens in response to different carbon sources, a reaction to sensing the presence of a plant in terms of cellulase expression with an involvement of YPR2 would not be without precendent. Moreover, *ypr2* transcript levels are subject to carbon source dependent regulation [13]. While a functional annotation of an alamethicin synthase is not available, the annotation of Druzhinina et al., 2016 [65] as supported by antismash analysis indicates TR_23171 for this function. In agreement with alamethicin levels (decreased to 23.8% of wildtype, 4.2 fold), our transcriptome data showed decreased transcript levels (4.3fold down in ∆*ypr2*) for the predicted alamethicin synthase gene *tex1*/TR_23171 [60] and hence supports the predicted function. Interestingly, alamethicin levels are also decreased in a strain lacking *sor5* (TR_73623; Fig. 4c), which is positively regulated by YPR2. It remains to be shown whether this regulation is direct or indirect and if it involves the function of SOR5.

The same screening also showed production of orsellinic acid by *T. reesei*, but only in constant darkness in QM6a and this metabolite was not detected in the absence of YPR2 or SOR5 (TR_73623). Presence of orsellinic acid in the wildtype was confirmed with three independent, subsequent sample sets. Therefore we aimed to identify the cluster responsible for orsellinic acid production in *T. reesei*. The closest homologue of the PKS encoding gene of the *A. nidulans* ors-cluster [66], *orsA* (ANID_07909), was found to be *T. reesei pks4* (TR_82208), which however represents the PKS responsible for pigment biosynthesis [67] and is related to the *wA* gene with the same function in *Aspergilli* [68]. Also a blast search with only the PksD domain (COG3321) yielded the same result. Accordingly, the whole *ors* cluster does not have direct homologues in *T. reesei* and *pks4* is not significantly regulated by YPR2.

Nielsen et al., [69] suggest a function for ANID_07903 in orsellinic acid biosynthesis. The homologue of this gene is TR_73621, which was recently shown to be involved in sorbicillin biosynthesis [5, 24]. However, deletion of TR_73621 has no significant influence on orsellinic acid production (data not shown) that would support such a function in *T. reesei*. The same study [69] reports detection of traces of orsellinic acid in strains lacking ANID_07903 and ANID_07909/orsA. These traces are attributed to unmethylated byproducts of the PKS ANID_08383 that produces dimethylorsellinic acid, but this PKS has no homologue in *T. reesei*.

Besides YPR2, also the monooxygenase TR_73623/SOR5 is required for orsellinic acid production in *T. reesei* (Fig. 4d) and deletion of *ypr2* strongly decreases *sor5* transcript levels in light and darkness [5]. The homologue of *sor5* in *A. nidulans*, ANID_07902, is located close to the ors cluster in the genome, but a connection to orsellinic acid has not been shown.

Using only the PksD domain of AN07909 (COG3321) for the homology analysis with *T. reesei*, we found again *pks4* (TR_82208) as best homologue, but another *pks* gene, TR_81694/*pks8g* with only marginally lower e-value and even higher identity with OrsA than PKS4 within this domain. Using the PksD domain of TR_81694 for a BLAST search against *A. nidulans* showed best homology to several PKSs other than OrsA, with highest score for PkgA. However, in contrast to *pks4*, TR_81694 is strongly down regulated in light and positively regulated by YPR2, which is in agreement with the levels detected for orsellinic acid. Additionally, three further genes within the cluster surrounding TR_81694 are coregulated and show light dependent downregulation and decreased transcript levels in ∆*ypr2*. AN7071/PkgA was found to be involved in production of several metabolites including alternariol [70] and the cluster in *T. reesei* is similar to that in *A. nidulans*.

These findings suggest that the biosynthesis of orsellinic acid in *T. reesei* is altered compared to *A. nidulans* and may involve the cluster around *pks8g*, which remains to be proven.

## Conclusions

Being a GRAS (generally regarded as safe) organism, production of potentially harmful metabolites is of particular interest with *T. reesei*. However, the findings in this field in the last years – connections between carbon and secondary metabolism and a regulatory relationship via the carbon catabolite repressor CRE1 [4, 5], even indicate a broad relevance for fungi in general. Detailed investigations of industrial strains and their products did not reveal production of harmful metabolites by *T. reesei*, indicating that induction mechanisms and/or structural genes are not operational or deleted in these strains.

A broader function of a transcription factor impacting enzyme expression as well as secondary metabolism was shown previously for *T. reesei* XPP1. This regulator was first described as an enzyme regulator [71], but later on found to have a much broader function, indicating that it might act as a switch between primary and secondary metabolism [4]. Also our early studies showed that the SOR cluster is regulated by CRE1 in a light dependent manner and that YPR2 is a regulator of the SOR cluster on cellulose [5], but seemed to have a broader function as well.

YPR2 influences gene regulation in darkness on cellulose considerably, indicating that the balance between carbon and secondary metabolism is highly relevant for *T. reesei*. In this respect it is also important to note that genes of the SOR cluster were among the most abundant upon growth under sexual development conditions [32]. Only recently, an inhibitory function of sorbicillinoids, which are produced by the SOR cluster gene products, on fungal pathogens was reported [72]. Moreover, although the transcript levels of the siderophore cluster regulated by YPR2 were low, consistent regulation of the whole cluster as well as coregulation of another siderophore associated NRPS (TR_60751) suggests siderophore regulation as a role for YPR2 as well. Siderophores are also known to play a role in competition and virulence in nature [56]. Consequently, regulation of the SOR cluster by YPR2 in response to environmental conditions may be crucial for successful competition and development.

Interestingly, our study now indicates that the two transcription factors YPR1 and YPR2 function in carbon source and light dependent regulation of the SOR cluster. With an enrichment of genes involved in glycolysis and glyconeogenesis (*p*-value 2.4e-03) in the gene set of upregulated genes in ∆*ypr2* in darkness (Additional file 2), also a connection of YPR2 to primary metabolism is supported. Upon growth on glucose, YPR2 represses the SOR cluster as well as *ypr1*, which is essential for the expression of the SOR cluster under these conditions [24]. In contrast, upon growth on cellulose, we found that YPR2 is a positive regulator of the SOR cluster [5] as well as of *ypr1*. Accordingly, *ypr1* levels are elevated on glucose, while *ypr2* levels are elevated on cellulose [13]. Additionally, the decreased levels of secondary metabolites upon growth in light on cellulose [5] are in agreement with our finding that in the wildtype, *ypr1* levels are strongly decreased in light. This carbon source dependent interplay of YPR1 and YPR2 supports the hypothesis of YPR2 being a regulator of primary, carbon and secondary metabolism in *T. reesei*.

In summary, our study revealed a broad, environment dependent function for YPR2 beyond the regulation of the secondary metabolite cluster in its genomic vicinity. Importantly, the considerable differences in gene regulation between light and darkness highlight the necessity of controlled light conditions for investigation of carbon and secondary metabolism in *T. reesei*.

## Methods

### Strains and cultivation conditions

*T. reesei* QM6a [8], ∆*ypr2* [5] and ∆*sor5* [5] were used throughout this study. Precultures for cultivation on cellulose were performed on plates with malt extract agar (3% *w*/*v*) in constant darkness for 14 days in order to avoid interference of light pulses or circadian rhythms with transcriptome analysis. An inoculum of 10^9^ conidia/L was applied to 100 ml of Mandels Andreotti minimal medium [73] with 1% (w/v) microcrystalline cellulose (Alfa Aesar, Karlsruhe, Germany) and 0.1% (w/v) peptone to induce germination. Strains were grown in constant light (1600 lx) or constant darkness at 28 °C for 72 h at 200 rpm. Harvesting of dark grown cultures was done under red safety light (darkroom lamp, Philips PF712E, red, 15 W) in order to avoid random light pulses and hence random gene regulation.

### Isolation of total RNA

Mycelia were harvested by filtration and frozen in liquid nitrogen. Total RNA was isolated essentially as described previously [74] using the QIAGEN plant RNA kit (QIAGEN, Hilden, Germany) according to manufacturer’s instructions. Quality control of total RNA was performed using Bioanalyzer 2100 (Agilent) and only high quality RNA (RIN-factor > 7.0) was used for further analysis.

### Transcriptome analysis and bioinformatics

RNA isolated from wildtype and ∆*ypr2* mutant after growth on cellulose in light and darkness was used for this analysis. We used two biological replicates for every condition and every strain. Next generation sequencing was performed at the core facility VetCORE (Vienna, Austria) on a HiSeq2000/SR50 machine. Per sample, 50–60 Mio reads were obtained and used for analysis. The mapping of reads was performed using bowtie2 with standard parameter setting [75]. The resulting files were further processed using samtools [76]. The comparison to the annotation was performed using bedtools [77]. FPKM-Values were calculated for each annotated transcript based on the results of the mapping (RSEM, [78]). Differential gene expression and statistically significant differences were evaluated using the software package edgeR [79]. For differential regulation a fold change threshold of 2 and a *p*-value of 0.01 was set. Generally the correlation between the two biological replicates used was very high, exceeding R = 0.975 in every case. Data are available at GEO (accession number GSE119126).

Hierarchical clustering was performed using the open source software HCE3.5 [80] which was used with default settings applying the Poisson correlation coefficient as the similarity/distance measure. Functional category analysis was done with the MIPS Functional Catalogue tool in the latest version available (May 2014; http://mips.helmholtz-muenchen.de/funcatDB/) [81].

### Biomass determination

Biomass determination in the presence of insoluble cellulose was performed as described previously [20]. Briefly, protein content of the biomass pellet reflecting biomass production of the fungus was analyzed by grinding the sample in liquid nitrogen, treatment with 0 .1N NaOH and sonication and determination of protein content by the Bradford method. At least two biological replicates were used.

### Analysis of secreted metabolites

Supernatants from cultures grown under similar conditions as for transcriptome analysis (minimal medium with cellulose as carbon source in light and darkness; see strains and cultivation conditions) were analyzed according to two previously described approaches. Depending on the experiment, two to seven biological replicates were used. Targeted measurements were carried out using a 5500 QTrap LC-MS/MS system (Applied Biosystems, Foster City, CA) as described in Sulyok et al. (2007) [82].

Targeted analysis for known compounds produced by fungi was performed essentially as described previously [5, 83]. This method has been extended and covers more than 700 metabolites at the moment. Calibration with serial dilutions of a multi analyte stock solution allows for reliable identification and quantification of secondary metabolites in the sample. Acquisition of two MRMs per analyte enabled confirmation of positive analyte identification. HPLC retention time as well as intensity ration of the two MRM transition was in agreement with the corresponding values of an authentic standard within 0.1 min and 30% rel., respectively.

These measurements were complemented by an untargeted metabolomics approach to screen for the global role of YPR2 in secondary metabolism of *T. reesei*. The native culture supernatants were measured by C_18_ reversed phase HPLC-HRMS on an LTQ Orbitrap XL system (Thermo Fisher Scientific, San Jose, USA) as described earlier [84]. In brief, 10-μL sample aliquots were injected into the LC-HRMS system and separated by linear gradient elution on an XBridge C_18_ column (150 × 2.1 mm i.d., 3.5 μm) (Waters, Milford, USA) at 25 °C. The mass spectrometer was equipped with an electrospray ionization source (positive ionization polarity) and operated in the fullscan mode (scan range m/z 100–1000) at a resolving power setting of 60.000 at m/z 400. Raw data files from the LC-HRMS measurements were centroided and converted to the open-data format mzXML with the ProteoWizard toolbox [85] and subsequently processed with the XCMS and CAMERA packages for untargeted metabolite detection [86, 87]. Briefly summarized, the XCMS and CAMERA package automatically detect chromatographic peaks in all data files, perform chromatographic alignment and integrate the metabolic features’ peak areas in the samples. The following parameters were adapted from their standard values for processing the dataset with XCMS and CAMERA: prefilter: 3 × 5000 counts, peak-detection algorithm: centwave, ppm: 5, peakwidth: max. 30 s, minfrac: 1, polarity used for metabolite annotation: positive. After chromatographic peak detection with XCMS and their annotation with the CAMERA package, we removed all peaks that were present in the blank samples as contaminants of non-fungal origin. Moreover, we manually removed several metabolite ion clusters originating from a biopolymer that caused high background levels. The remaining features (i.e. chromatographic peaks) were used for statistical analysis of the dataset. For this, the peak areas of the fungal features from the untargeted metabolomics data processing were first normalized by their determined biomass. Then we illustrated the dataset in the form of a bi-clustered heatmap. For this, the peak areas of the features were mean-centered and auto-scaled. The dendrograms for the heatmap were generated using squared Euclidean distance and ward-linkage. The dendrogram of the features was subsequently manually split into 6 groups and boxplots were generated for the feature areas of the respective metabolites in the respective group in order to illustrate the abundance levels of the different metabolites in the biological samples.

## Additional files

Additional file 1: Gene regulation by YPR2. This file contains data on gene regulation by YPR2 in constant light and constant darkness upon growth on cellulose along with clustered regulation and overlapping regulation with induction specific genes as well as by photoreceptors and CRE1. (XLSX 721 kb)

Additional file 2: Functional category analysis of YPR2 targets. (XLSX 105 kb)

Additional file 3: **Figure S1.** Showing functional categories of YPR2 targets in light. (PDF 300 kb)

Additional file 4: Abundance of secondary metabolites as influenced by YPR2. This file contains mass spectrometry data from analysis of wildtype and ∆*ypr2* in light and darkness. (XLSX 212 kb)

## Abbreviations

HPLC: High performance liquid chromatography
LC-MS: Liquid chromatography mass spectrometry
NRPS: Non ribosomal peptide synthase
RIN factor: RNA integrity factor
