# Supplementary material for: YPR2 is a regulator of light modulated carbon and secondary metabolism in Trichoderma reesei
Source: BMC Genomics. 2019 Mar 13;20:211. doi: 10.1186/s12864-019-5574-8 (PMC6417087; doi:10.1186/s12864-019-5574-8)
Supplement: Supplementary file 3 — Figure S1. Showing functional categories of YPR2 targets in light. (PDF 300 kb) [file 12864_2019_5574_MOESM3_ESM.pdf]

YPR2 is a light dependent regulator of carbon and secondary metabolism in *Trichoderma reesei*

SUPPLEMENTARY MATERIAL

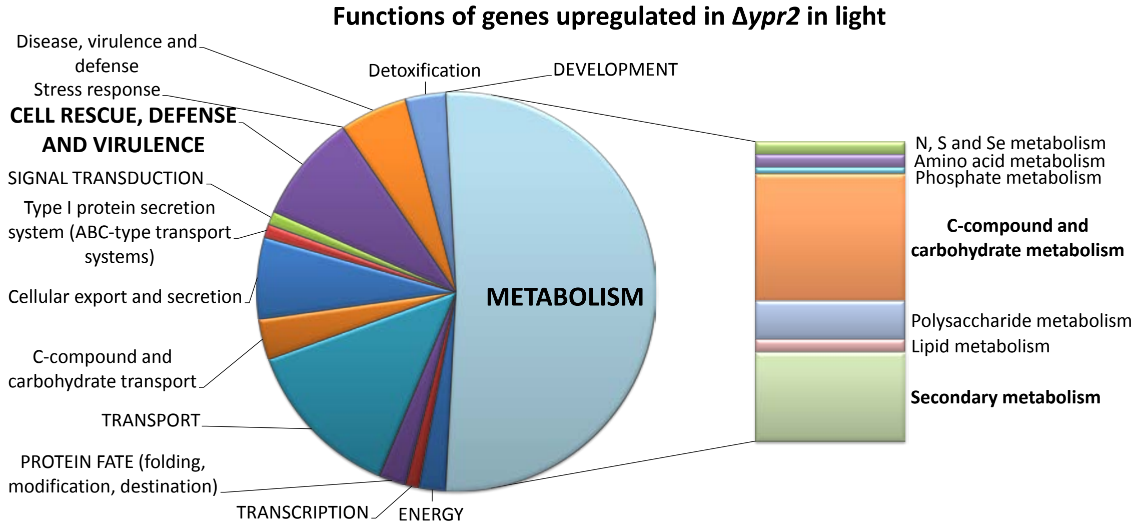

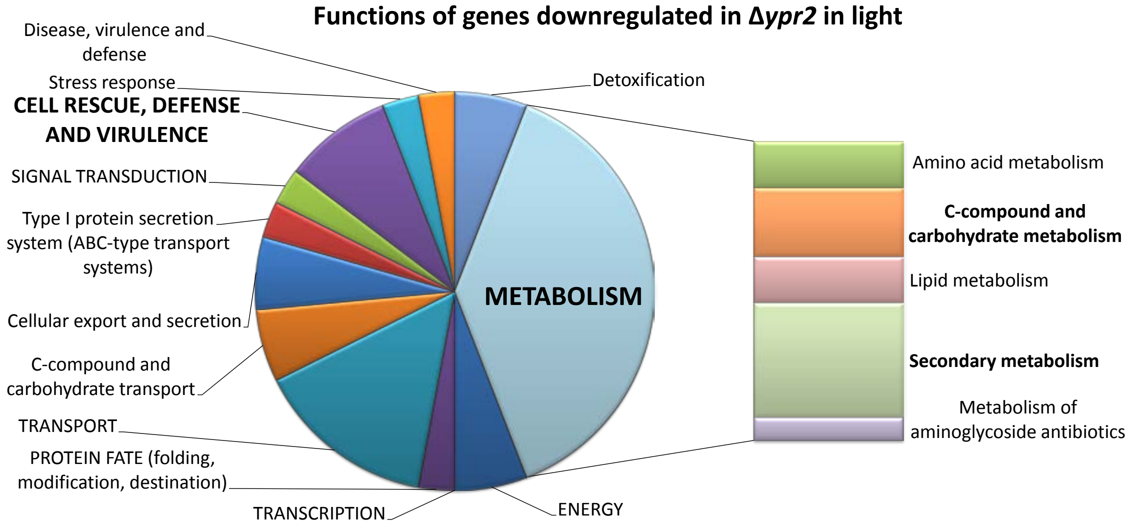

Figure S1. Functions of genes regulated in Δ*ypr2* compared to wildtype upon growth on cellulose in light.
